# Population Health Inequalities Across and Within European Metropolitan Areas through the Lens of the EURO-HEALTHY Population Health Index

**DOI:** 10.3390/ijerph16050836

**Published:** 2019-03-07

**Authors:** Claudia Costa, Paula Santana, Sani Dimitroulopoulou, Bo Burstrom, Carme Borrell, Jürgen Schweikart, Dagmar Dzurova, Nicolás Zangarini, Klea Katsouyanni, Patrick Deboseree, Ângela Freitas, Christina Mitsakou, Evangelia Samoli, Sotiris Vardoulakis, Marc Marí Dell’Olmo, Mercè Gotsens, Michala Lustigova, Diana Corman, Giuseppe Costa

**Affiliations:** 1 Centre of Studies in Geography and Spatial Planning, University of Coimbra, 3004-530 Coimbra, Portugal; paulasantana.coimbra@gmail.com (P.S.); angelafreitas30@gmail.com (Â.F.); 2 Department of Geography and Tourism, University of Coimbra, 3004-530 Coimbra, Portugal; 3 Centre for Radiation, Chemical and Environmental Hazards, Public Health England, Chilton OX11 0RQ, Oxon, UK; Sani.Dimitroulopoulou@phe.gov.uk (S.D.); Christina.Mitsakou@phe.gov.uk (C.M.); 4 Karolinska Institutet, Department of Public Health Sciences, Division of Social Medicine, 171 77 Stockholm, Sweden; bo.burstrom@sll.se; 5 Agència de Salut Pública de Barcelona, 08023 Barcelona, Spain; cborrell@aspb.cat (C.B.); mmari@aspb.cat (M.M.D.); mgotsens@aspb.cat (M.G.); 6 CIBER Epidemiología y Salud Pública (CIBERESP), 28029 Madrid, Spain; 7 Institut d’Investigació Biomèdica (IIB Sant Pau), 08041 Barcelona, Spain; 8 Universitat Pompeu Fabra, 08002 Barcelona, Spain; 9 Department of Civil Engineering and Geoinformation, Beuth University of Applied Sciences Berlin, 13437 Berlin, Germany; schweikart@beuth-hochschule.de; 10 Department of Social Geography and Regional Development, Faculty of Science, Charles University, 128 43 Prague, Czech Republic; dagmar.dzurova@natur.cuni.cz (D.D.); michala.lustigova@gmail.com (M.L.); 11 Department of Public Health and Pediatrics, University of Turin, 10126 Turin, Italy; nicolas.zengarini@epi.piemonte.it; 12 Department of Hygiene, Epidemiology and Medical Statistics, University of Athens Medical School, 115 27 Athens, Greece; kkatsouy@med.uoa.gr (K.K.); esamoli@med.uoa.gr (E.S.); 13 Interface Demography, University of Brussels, 1050 Brussels, Belgium; patrick.deboosere@vub.ac.be; 14 Institute of Occupational Medicine, Edinburgh EH14 4AP, UK; Sotiris.Vardoulakis@iom-world.org; 15 The National Board of Health and Welfare, 106 30 Stockholm, Sweden; diana.corman@socialstyrelsen.se; 16 Medical School of the University of Turin, University of Turin, 10124 Turin, Italy; giuseppe.costa@epi.piemonte.it

**Keywords:** Population Health Index, Europe, metropolitan areas, health determinants, health outcomes, municipalities

## Abstract

The different geographical contexts seen in European metropolitan areas are reflected in the uneven distribution of health risk factors for the population. Accumulating evidence on multiple health determinants point to the importance of individual, social, economic, physical and built environment features, which can be shaped by the local authorities. The complexity of measuring health, which at the same time underscores the level of intra-urban inequalities, calls for integrated and multidimensional approaches. The aim of this study is to analyse inequalities in health determinants and health outcomes across and within nine metropolitan areas: Athens, Barcelona, Berlin-Brandenburg, Brussels, Lisbon, London, Prague, Stockholm and Turin. We use the EURO-HEALTHY Population Health Index (PHI), a tool that measures health in two components: Health Determinants and Health Outcomes. The application of this tool revealed important inequalities between metropolitan areas: Better scores were found in Northern cities when compared with their Southern and Eastern counterparts in both components. The analysis of geographical patterns within metropolitan areas showed that there are intra-urban inequalities, and, in most cities, they appear to form spatial clusters. Identifying which urban areas are measurably worse off, in either Health Determinants or Health Outcomes, or both, provides a basis for redirecting local action and for ongoing comparisons with other metropolitan areas.

## 1. Introduction

Health is a critical global development issue, especially in urban areas where the majority of the world’s population lives [1,2,3,4]. The United Nations Sustainable Development Goals (SDG) put a focus on health promotion through a number of interconnected health-related targets like SDG-3 (good health and well-being), SDG-10 (reduced inequalities) and SDG-11 (sustainable cities and communities). These goals are achievable through multisectoral approaches, as stated in the New Urban Agenda [5,6,7]. There is ample evidence that contextual factors related with the social, physical and built urban environments affect health and are key drivers of health inequalities within cities: The access to green spaces and public places, the exposure to air pollution and noise, the access to affordable housing, the opportunity to use public transportation, to walk and bike, among others [6,8,9,10,11,12,13,14,15].

The main factors influencing the overall health of the population are well illustrated by Dahlgren and Whitehead’s model of social determinants of health [16,17], which describes the different layers of influence: Individual lifestyle factors, social and community networks, living working and conditions and general socioeconomic, cultural and environmental conditions in which the population lives. The determinants of health are shaped by individual and political decisions and can be either positive health factors (e.g., economic security, adequate housing), protective (e.g., social support, healthy diet) or risk factors (e.g., pollution, smoking) [17]. The recent study on environmental public health indicators in European urban areas, within the framework of the EURO-HEALTHY project, explored this through the association between the health impacts and the physical and built environmental risks in order to support the prioritisation of interventions that improve public health and reduce avoidable deaths [18].

There is a growing body of evidence showing a strong connection between the characteristics of the place of residence and health outcomes, even after accounting for individual risk factors [8,13,18,19,20,21,22,23,24,25]. High levels of intra-urban inequalities are also visible as a result of the demographic, economic, environmental, and other societal challenges impacting cities, along with a greater population heterogeneity and different level of access to housing, amenities and services [2,10,13,26]. According to the literature, poor and vulnerable groups are often more at risk due to the concentration on disadvantaged and deprived neighbourhoods, usually in the outskirts of the city or in inner city areas [27,28,29,30]. The Atlas of Population Health in European Union Regions [31], shows that the capital region of each EU country ussually performs better in health determinants (e.g., economic and social conditions, healthcare resources) than in health outcomes. Capital regions are often affected by increased levels of crime and air pollution with negative impacts on health outcomes. Growing evidence demonstrates an excess on mortality and greatest burden of disease on urban areas compared to non-urban, namely related with lung cancer and cardiovascular disease [32,33], and of greater relative socioeconomic inequalities in mortality in Eastern and Northern European cities, although with variations in their magnitude [34,35].

A deeper understanding of the interconnection between compositional and contextual factors and how they affect health outcomes is required of local decision makers in order to better cope with the complexity of addressing health determinants that goes beyond traditional behavioural change approaches [2,13,20,22,36]. Most of the policy interventions largely concentrate on modifying the midstream determinants of health, the intermediate factors, such as individual health behaviours (smoking, physical activity, nutrition) and on targeting vulnerable groups to mitigate the negative impacts of disadvantage on health. Policies and actions need to be directed towards improving fundamental social and economic structures in order to remove barriers and allow people to achieve their full health potential [37,38,39,40]. Local governments, especially those from urban areas, occupy a unique leadership position on levelling up policies to tackle determinants of health, working across the upstream, midstream and downstream levels [1,25,40,41].

To effectively address the causes of health inequalities, spatially disaggregated data on different health determinants and better urban health metrics are needed [42,43]. A measure that summarises crucial data provides opportunities to understand the complexity of how much health differs within and across urban areas, given how it offers a comprehensive picture of health and health disparities. Nevertheless, the ability to include meaningful information into a single metric that captures the level of health, the intensity of health determinants, and the extent of disparities, is limited [44].

It was within the scope of the EU-funded project EURO-HEALTHY (shaping European policies to promote health equity) whose aim was to advance the knowledge on policies with the highest potential to promote health equity, that a multidimensional and multilevel index—the Population Health Index (PHI)—was built. This measure evaluates European population health across a wide range of areas of concern, dimensions and indicators of health determinants and health outcomes [31]. Its construction integrates the technical elements of a multi-criteria value model and the social elements of interdisciplinary and participatory processes by collecting the views of experts and stakeholders on which factors are relevant to evaluate health [45] and on how important it is to close the gap between indicators to improve population health [46].

More than 75% of the European population lives in urban areas, thus reflecting and encompassing a diversity of geographies and of physical, social, and economic environments. By using the EURO-HEALTHY Population Health Index (PHI), the aim of this paper was to identify inequalities in health determinants and health outcomes across and within nine European metropolitan areas.

## 2. Materials and Methods

### 2.1. Study Area

We applied an ecological study to analyse the Health Determinants and Health Outcomes value-scores between and within metropolitan areas, taking 2014 as a reference year.

The indicators were collected at two levels defined by EUROSTAT [47]: Local administrative units (LAU) 1, corresponding to small areas, and LAU 2, corresponding to municipalities. The utilisation of both levels is explained by the diversified system of local governments in Europe and the pre-existent request from the EURO-HEALTHY project to employ the PHI to a political subdivision where a local government may implement interventions able to address health inequalities. Each metropolitan area specified different administrative levels, most of them corresponding to municipalities. Additional information on the LAU and delimitation of each metropolitan area is provided in Supplementary Materials S1.

**Figure 1:**
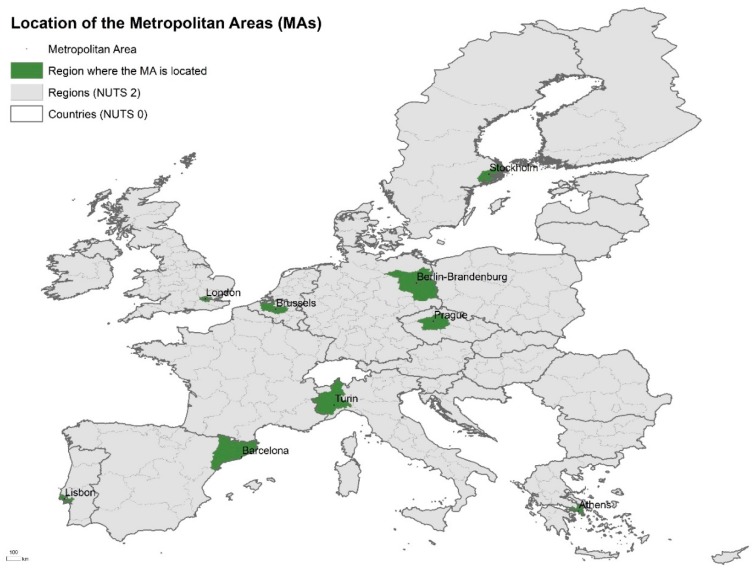
Location of the nine metropolitan areas selected. NUTS: Nomenclature of Territorial Units for Statistics.

The study area corresponds to 328 administrative areas from the nine metropolitan areas located in different geographical regions in Europe: Athens, Greece (40 LAU 1); Barcelona, Spain (23 LAU 2); Berlin-Brandenburg, Germany (23 LAU 1 and 2); Brussels, Belgium (91 LAU 2); Lisbon, Portugal (18 LAU 1); London, United Kingdom (33 LAU 1); Prague, Czechia (25 LAU 1 or 2), Stockholm, Sweden (26 LAU 2) and Turin, Italy (49 LAU 2) (Figure 1).

The nine metropolitan areas analysed in this study where selected under the EURO-HEALTHY project framework. These areas represent the different EU geographic zones and populations (Northern, Southern, Central and Eastern Europe) reflecting Europe’s diversity—in terms of contextual conditions (e.g., geographical, historical, political, cultural, social and economic) and impact of the financial and economic crisis (Table 1).

### 2.2. Applying the Population Health Index

The measure used in this study is the EURO-HEALTHY PHI, which was built to evaluate population health of the EU regions for the reference year 2014. Presenting a bottom-up hierarchical structure with several indices, the PHI measures health with respect to the components Health Determinants and Health Outcomes, both disaggregated into areas of concern, dimensions and indicators (Table 2) [31]. The Health Determinants component represents the contextual factors defined as the environmental conditions in which people live and which directly and/or indirectly influence health: Economic conditions, social protection and security, education, demographic change, lifestyle and health behaviours, physical environment, built environment, road safety, healthcare resources and expenditure, and healthcare performance. The Health Outcomes component refers to the severity and frequency of disease and/or death, including both mortality and morbidity indicators.

A socio-technical approach was developed by the EURO-HEALTHY team to build the PHI. The methodology combines the multi-criteria MACBETH method [48] which included several participatory processes, namely Web-Delphi processes and a decision conferencing process. Creating the PHI involved two main phases: The first, which identified and defined the areas of concern, dimensions, and indicators considered relevant to evaluate population health [45] and the subsequent evaluation phase, where qualitative value judgments were elicited from the panel of experts and stakeholders on the weighting of indicators and the shape of the value function for each indicator. Driving the discussions were considerations on the importance of closing the gaps of performance in the indicators and of the added value of improvements in each indicator, with a view toward reducing health inequalities [46]. Experts and stakeholders, representing regional and local contexts and with a multidisciplinary background and expertise, were involved throughout all the phases of the PHI’s construction [49].

The conceptual and methodological approach used to model the EURO-HEALTHY PHI (applied to the 269 regions of the EU 28 countries) was adjusted to the nine metropolitan areas regarding the structure, weights and value functions [31]. A score was calculated for each component, area of concern, dimension and indicator allowing the comparison of population health between geographical areas in an aggregated or disaggregated way. The value-scores ranged from 0 to 100, where 0 represented the lowest score of population health and 100 the highest score. The colour coding of the classes uses a gradation inspired by a traffic system: Red representing low values and green colours representing high scores.

#### Indicators

39 indicators where selected for inclusion in the PHI and framed by component, area of concern and dimension (Table 2) [31]. From these, 26 indicators were requested to be collected at the municipal level. The data collection process followed six steps: (a) Identification of a focal point (designated researcher) in each metropolitan area responsible for the data collection; (b) application of a survey to the focal points to identify the availability of the indicators at local level; (c) selection of the indicators to be collected at the local level; (d) production of a manual detailing how to build and deliver the indicators; (e) data collection and processing of the data; and (f) delivery performed through a web platform with data quality procedures.

The data availability of the PHI indicators in each metropolitan area, data source and year of the data is provided in Supplementary Materials S2.

### 2.3. Statistical Analysis

In order to provide an integrated description of population health inequalities across and within metropolitan areas, three main analyses were performed.

An Analysis of Variance (ANOVA) was applied to detect whether there were statistically significant differences in the PHI scores of the Health Determinants and Health Outcomes components across the metropolitan areas. The procedure works by comparing the variance between pairs of metropolitan areas means versus the variance within metropolitan areas as a way of determining whether there are similarities or disparities. Scheffe’s test was used at a statistical significance level of 0.05. The analysis was performed using SPSS software (IBM, Armonk, NY, USA).

The coefficient of variation (CV) was calculated to measure dispersion on value-scores for both Health Determinants and Health Outcomes Indices across municipalities of the same metropolitan area. The smaller the CV value, the greater the data homogeneity and the smaller the variation. The analysis was performed using Excel software (Microsoft, Redmond, WA, USA).

Finally, the LISA (Local Indicator of Spatial Association) measure was used to identify local patterns of spatial association between spatial units (municipalities), upon confrontation with their neighbours. This spatial correlation method allowed the identification of geographical clusters with identical values, defined by the spatial concentration of low scores (Low-Low) and high scores (High-High). The analysis was performed using ArcGIS software (Esri, Redlands, CA, USA).

## 3. Results

When the EURO-HEALTHY PHI was applied to the metropolitan areas, a geographical variation in the distribution of the value-scores was revealed across metropolitan areas in both components. Overall, almost all the municipalities registered value-scores above 50 (the PHI ranges from 0 to 100), with 31% attaining 75 and above in both components.

Table 3 and Table 4 presents the results of the pairwise comparisons of metropolitan areas with respect to Health Determinants and Health Outcomes Indices. Four groups of metropolitan areas emerge: (1) Stockholm stands out with a significantly higher mean score in both components (above 87 on both components); (2) Athens, Barcelona and Lisbon present lower values in Health Determinants (below 62.7); (3) Barcelona and Turin present high scores in Health Outcomes (around 83); and (4) Lisbon and Prague present lower scores in Health Outcomes (below 66.7).

**Figure 2:**
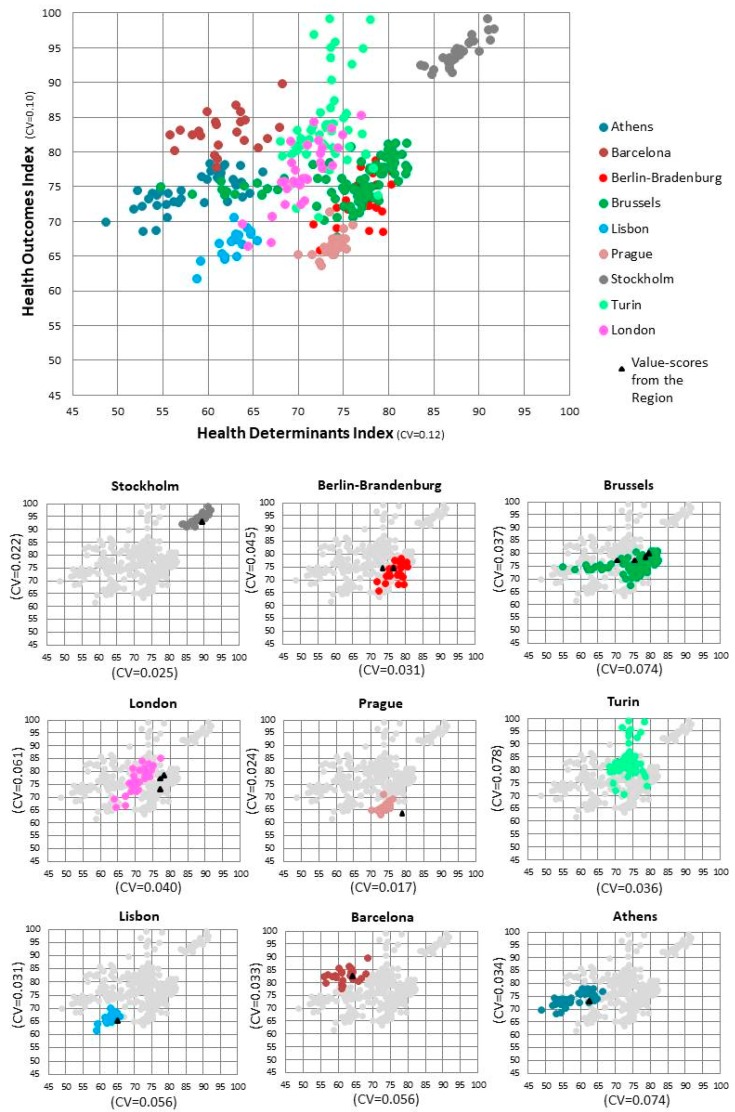
Scatterplot of the Health Determinants and Health Outcomes value-scores by metropolitan area and corresponding coefficient of variation (CV). Note: Each colour represents one metropolitan area and each dot a municipality. The triangle represents the value-scores from the region (NUTS 2 level) where the metropolitan area is located. The coordinates of the dots and triangles are based on the value-score achieved by the municipality/region on the Health Determinants Index (x-axis) and on the Health Outcomes Index (y-axis).

Figure 2 presents a 12% variation in the Health Determinants Index (CV = 0.12) and a 10% variation in the Health Outcomes Index (CV = 0.10). Although the variation is lower when we analyse each metropolitan area, higher internal variability was identified for Brussels and Athens in Health Determinants (CV ≥ 0.074), whereas in Health Outcomes, the same variability was found in Turin and London (CV ≥ 0.061).

In addition, this figure reveals that 55% of the municipalities in this study perform better in Health Determinants than the region where they are located [31]. Exceptions are found in Berlin-Brandenburg, Brussels and Turin, where more than 60% of the municipalities present worse values. In Health Outcomes, the opposite was found. Berlin-Brandenburg and Brussels are the exceptions, where more than 70% of the municipalities present better values. Prague stands out since all the municipalities perform better than the region in Health Determinants and worse in Health Outcomes.

**Figure 3:**
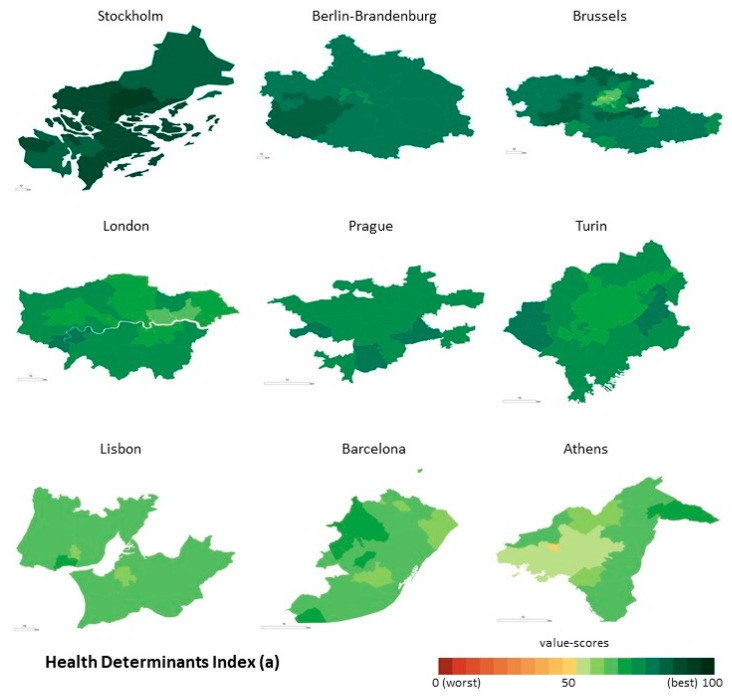

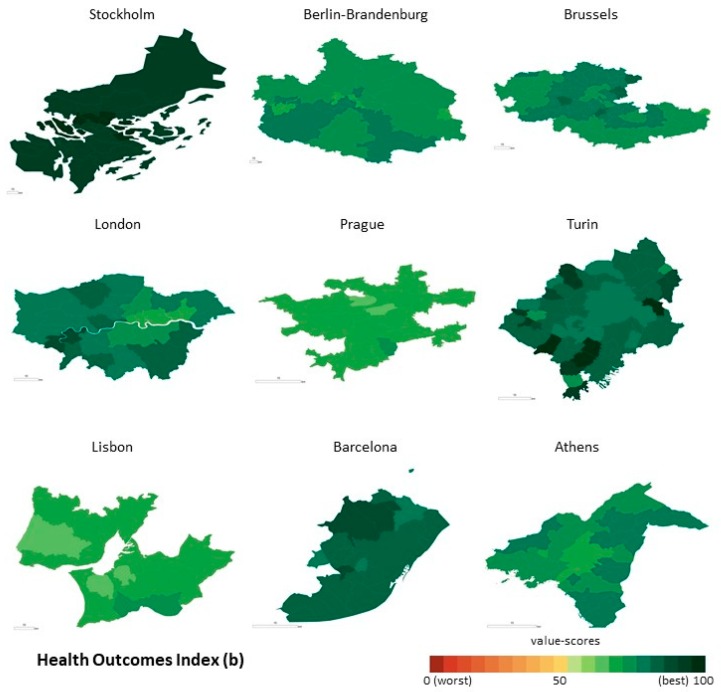
Geographical distribution of the PHI on the Health Determinants Index (**a**) and on the Health Outcomes Index (**b**), at the municipal level. Note: The value-scores are displayed by using classification by Equal interval, taking into account the PHI minimum and maximum scores (from 0 to 100). The colour coding of the classes used a gradation inspired by a traffic light system (from red to green). In the case of the metropolitan areas, the light green represents the municipalities with worse population health and dark green represent better scores.

The distribution of Health Determinants is not homogeneous across and within the metropolitan areas (Figure 3a). There is a gradient from northern European to southern European countries, with higher scores being found in Stockholm, Berlin and Brussels and lower scores in Lisbon, Athens and Barcelona. When looking at the within-metropolitan areas inequalities, the geographical pattern differs when we compare scores from a centre-periphery model point of view. The metropolitan centre of Brussels and Berlin-Brandenburg present lower scores when compared with the municipalities located in the periphery. The opposite is found in Stockholm.

As for Health Outcomes, the north-south gradient is not evident (Figure 3b). Along with Stockholm, Turin and Barcelona registered higher scores. Lower scores were identified in Lisbon and Prague. The geographical variation in the distribution of the value-scores across municipalities is considerable when compared to Health Determinants, with no clear pattern being found in the majority of the metropolitan areas. However, it is visible that the centres from Brussels, Athens and Berlin-Brandenburg present lower value-scores than the periphery.

**Figure 4:**
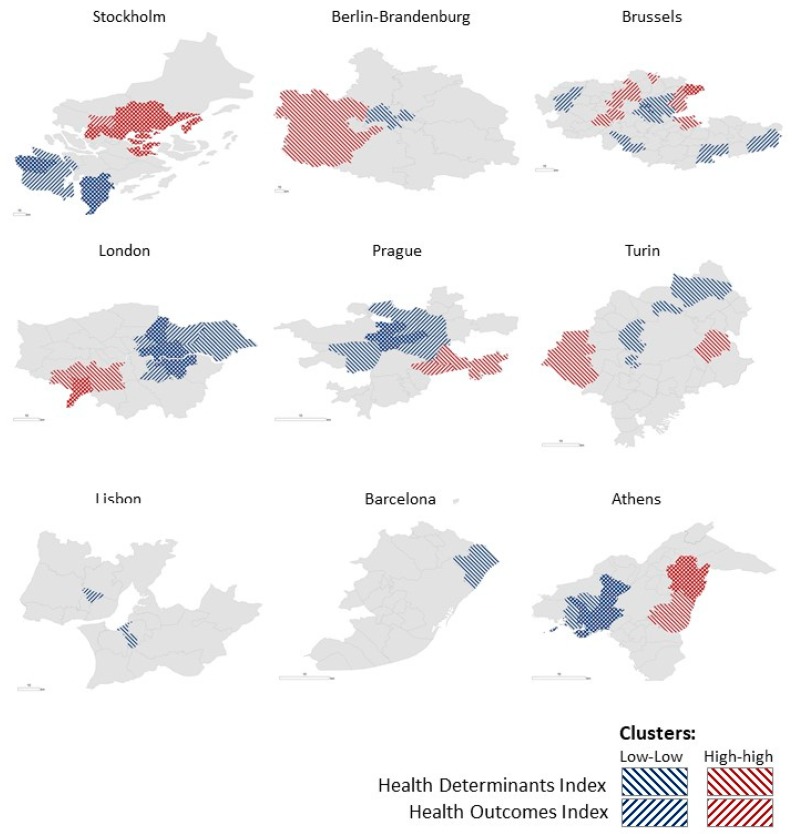
Clusters of municipalities within the Health Determinants and the Health Outcomes Indices. Note: The figure represents the clusters identified for both the Health Determinants Index (backward diagonal shading) and the Health Outcomes Index (forward diagonal shading). Blue lines represent the municipalities with low value-scores that are surrounded by municipalities also with low value-scores (cluster Low-Low). Red lines represent the municipalities with high value-scores that are surrounded by municipalities which also register high value-scores (cluster High-High).

Figure 4 illustrates the presence of clusters in all metropolitan areas and in both components, apart from Barcelona, Turin and Berlin-Brandenburg where they were only identified for the Health Determinants Index. Almost 1/4 of the population being studied lives in municipalities located in the Low-Low clusters (concentration of lower value-scores) in Health Determinants while only 8% are living in clusters characterised by a concentration of higher value-scores (High-High). For the Health Outcomes Index, the rates are also relevant:11% are clustered in Low-Low and 4% are clustered in High-High. The analysis also revealed populations living in municipalities classified in the cluster Low-Low for both Health Determinants and Health Outcomes indices in Athens (17%), Prague (16%), London (13%), Brussels (7%) and Stockholm (2%). In Brussels (0.5%), London (2%), Athens (8%) and Stockholm (21%), there are also municipalities classified in the cluster High-High for both indices.

## 4. Discussion

The objective of this study was to analyse health inequalities as measured by Health Determinants and Health Outcomes indices, across and within different European metropolitan areas.

The results contribute to deepening the knowledge about health at the urban level and follow previous work done across European regions [31] under the scope of the EURO-HEALTHY project. In the application of the Population Health Index (PHI), designed to evaluate health in two multidimensional components, Health Determinants and Health Outcomes, this study examines the results obtained from nine metropolitan areas (Athens, Barcelona, Berlin-Brandenburg, Brussels, Lisbon, London, Prague, Stockholm and Turin) which represent different European regions and heterogeneous geographic, social and economic contexts.

Overall, it was found that: (i) Strong population health inequalities exist across metropolitan areas, with municipalities from Southern and Eastern countries presenting, in general, worse value-scores; (ii) metropolitan areas present better health, measured by Health Determinants, than the region where they belong, although some exceptions were found, and; (iii) Municipalities with worse Health Determinants scores tend to also perform worse on Health Outcomes.

Thus, the analysis of the distribution of the value-scores on both indices shows a high dispersion across metropolitan areas: In Health Determinants the range goes from 49 (Athens) to 92 (Stockholm) and in Health Outcomes, from 62 (Lisbon) to 99 (Stockholm). The fact that urban areas from North-Western countries present better health scores than the Eastern and Southern ones is not new and is aligned with results from previous studies on population health in Europe [31,35,50].

Simultaneous to a high difference in value-scores among metropolitan areas, there is a considerable variation within municipalities of the same metropolitan area. Of note are Brussels and Athens, which display a clear geographic variation in Health Determinants scores, and Turin and London in Health Outcomes. Previous studies also identified the presence of inequalities within these metropolitan areas, although at a more detailed scale [29,35,51]. For example, in the Lisbon case, geographic disparities between municipalities are not evident as expected, considering other studies on health inequalities at the small area level [19,21,27].

As the PHI model was previously applied to EU regions, it offered the possibility to compare the population health scores of metropolitan areas to those performed by the respective regions where they are located. In opposition to what it was identified for the regions, the municipalities from the metropolitan areas often perform better on Health Outcomes than on Health Determinants. Nonetheless, it was found that most municipalities performed better scores in the Health Determinants index when compared with the regional scores. Prague stands out as a paradigmatic case: All municipalities present significantly better scores than the respective region in Health Determinants, performing worse in Health Outcomes. These results may be understood as ‘ambiguous’, considering that the country and specifically the capital were emerging from a long-period of social and economic stagnation and recession, with negative impacts on health determinants [52,53]. The contrary occurs with Berlin-Brandenburg, Brussels and Turin, considering that these metropolitan areas present worse Health Determinants scores than their respective regions. One plausible explanation is that when they are compared with the larger administrative region, they perform worse in important health determinants, such as high levels of air pollution, ageing and crime, indicators used to build the PHI. According to another recent study from the EURO-HEALHTY project, using data from the same nine metropolitan areas, revealed that worse air quality is typically encountered in deprived European urban areas [54]. Still, those health determinants do not affect those metropolitan areas equally. Brussels, for instance, is younger than the rest of the country [55]. Unemployment and poor housing conditions provide a better explanation for this metropolitan area [56].

The application of LISA to detect spatial concentrations of similar scores within the same metropolitan area revealed that the share of the population living in Low-Low clusters (concentration of lower value-scores) in Health Determinants is three times higher than those living in clusters characterized by a concentration of higher value-scores (High-High cluster). Also, in a considerable number of urban areas, clusters were revealed in both indices. This is the case of Brussels, London, Athens and Stockholm, with clusters of High-High and Low-Low value-scores for both indices. The municipalities that share worse health determinants and worse health outcomes (Low-Low clusters) should be pointed out as ‘urban zones in alert’ calling the attention of local policy makers to the need to address population health in an inter-sectoral and integrated way.

Measuring health at the local level is complex since there are diverse and interconnected factors operating at different scales in the same place [57]. The result of the application of a single index to depict inequalities is not immune to criticism and should be interpreted considering some limitations. Though the greatest asset of having aggregated indices is the simplification of data and the possibility to compare different geographical units using a single measure, there is always an amount of information that is lost [58]. Thus, some aspects regarding the administrative delimitation of metropolitan areas and the type of indicators used in the PHI model may contribute to masking inequalities. The lack of consensus on the delimitation of the metropolitan areas led to the utilization of different functional definitions and administrative levels. Although this issue was already referred to in previous studies as a limitation of ecological studies in Europe, so far, there remains no solution [28,29,34,35]. Moreover, the population size of municipalities within the same metropolitan area (e.g., Barcelona has a municipality with more than 1.5 M inhabitants and other with less than 200,000) can introduce an important MAUP (Modifiable Areal Unit Problem) effect [59].

According to Rothenberg and others [44], inequality among countries is mirrored in the inequality within their regions and cities. Therefore, bottom-up approaches based on local data and knowledge is of high relevance to promoting equity-based policies [1,10,36,40,60,61,62]. The type of indicators used in the construction of urban health indices is key to detect inequalities among municipalities. The Health Determinants and Health Outcomes indices analysed in this study are the result of an aggregation of multiple dimensions and indicators that were selected as relevant to evaluate population health at a regional level. This participatory process, conducted with stakeholders representing the countries of these metropolitan areas and the indicators selected, was analysed by Freitas and others [45]. Although indicators had been selected for a regional scale, they were considered as adequate and reliable proxies to measure health determinants and health outcomes at the metropolitan level. Yet, indicators focusing on specific urban characteristics (e.g., access to green spaces, transportation, social protection) and more sensitive to the local social, physical and built environment [6,8,9,10] are not included in the PHI, not because they were not considered relevant, but due to the lack of availability and/or comparability across regions, two criteria required for an indicator to be included in the PHI [45,63].

The application of an urban health index to the municipalities of nine European metropolitan areas, very different in contexts and levels of data availability, brought some constraints and represented some risks, implying the need to apply a predefined and common protocol of data collection and harmonization. The lack of indicators at the administrative level of the municipality led to the use of data at coarser geographical resolution—regional or national. This indicates that besides the need to reinforce the data collection at the sub-national level, already identified in previous studies [31,63], there are different levels of capacity from national and municipal statistics to collect urban health-related data at the local level.

Regardless of the above-mentioned limitations, the strength of this study is to show that geographical analysis is needed when investigating health inequalities. These results should be understood as a point of departure to show that there are inequalities within the same region and metropolitan area and there is a need to examine and act locally to address any existing inequities. And though we focused only on aggregated indices of Health Determinants and Health Outcomes, it offers enough clues that a multi-sectoral commitment between the health sector and other sectors at the local level would be valuable when it comes to promoting population health.

## 5. Conclusions

This study adds evidence to the debate on the existence of health inequalities across Europe: Not only across countries and regions, but also among and within urban areas.

The application of a single measure—the Population Health Index—to evaluate health determinants and health outcomes in nine European metropolitan areas show that not only do they exhibit differences between them, but that municipalities within the same urban area face different population health profiles. Conversely, municipalities from these nine metropolitan areas have now a common tool to compare themselves with and share the lessons on how to tackle similar health problems.

The responsibility of promoting health does not lie exclusively in the health sector or with the national government. More and more, municipalities across Europe are demonstrating their responsability when it comes to adopting policies that improve the health and well-being of their citizens.

## Figures and Tables

**Table 1:** General characteristics of the metropolitan areas.

Metropolitan Area	Area (km^2^)	Density (Inhabitants/km^2^)	Population (Inhabitants)	Population +65 (%)	Population Range (Inhabitants)
Athens	403	7669	3,090,508	17.8	25,389–664,046
Barcelona	420	7590	3,103,973	18.9	13,531–1,611,822
Berlin—Brandenburg	16,669	352	5,871,022	20.7	58,018–371,438
Brussels	3591	698	2,504,715	16.1	2160–176,545
Prague	315	3737	1,177,141	18.5	6021–128,063
Lisbon	2917	966	2,821,876	18.2	17,569–547,733
London	1468	5733	8,416,543	11.4	7648–372,752
Stockholm	6011	348	2,091,449	15.2	9331–864,315
Turin	1000	1620	1,619,478	24.8	1200–886,837

**Table 2:** List of the EURO-HEALTHY Population Health Index (PHI).

Component: Health Determinants		
Area of Concern	Dimension	Indicator
Economic conditions, social protection and security	Employment	Unemployment rate (%)
		Long-term unemployment rate (%)
	Income and Living conditions	Disposable income of private households per capita (Euro per inhabitant)
		People at risk of poverty or social exclusion (%)
		Disposable income ratio—S80/S20
	Social protection	Expenditure on care for the elderly (% of GDP)
	Security	Crimes recorded by the police (per 100,000 inhabitants)
Education	Education	Population aged 25–64 with upper secondary or tertiary education attainment (%)
		Early leavers from education and training (%)
Demographic change	Ageing	At risk of poverty rate of older people (%)
		Ageing index (ratio)
Lifestyle and health behaviours	Lifestyle and health behaviours	Adults who are obese (%)
		Daily smokers—aged 15 and over (%)
		Pure alcohol consumption—aged 15 and over (Litres per capita)
		Live births by mothers under age of 20 (%)
Physical environment	Pollution	Annual mean of daily PM_2.5_ concentrations (ug/m^3^)
		Annual mean of daily PM_10_ concentrations (ug/m^3^)
		Greenhouse Gas (total tonnes of CO_2_ eq. emissions per capita)
Built environment	Housing conditions	Average number of rooms per person
		Households without indoor flushing toilet (%)
		Households without central heating (%)
	Water and sanitation	Population connected to public water supply (%)
		Population connected to wastewater treatment plants (%)
	Recycling	Recycling rate of municipal waste (%)
Road safety	Road safety	Victims of road accidents—injured and killed (per 100,000 inhabitants)
		Fatality rate due to road traffic accidents (per 1000 victims)
Healthcare resources and expenditure	Healthcare resources	Medical doctors (per 100,000 inhabitants)
		Health personnel—nurses and midwives, dentists, pharmacists and physiotherapists (per 100,000 inhabitants)
	Healthcare expenditure	Total health expenditure (Purchasing Power Parity per capita)
		Private households’ out-of-pocket expenses on health (% total health expenditure)
		Total health expenditure (Purchasing Power Parity per capita)
Healthcare performance	Healthcare performance	Hospital discharges due to diabetes, hypertension and asthma (per 100,000 inhabitants)
		Amenable deaths due to healthcare (standardized death rate per 100,000 inhabitants)
**Component: Health Outcomes**		
Health Outcomes	Morbidity	Self-perceived health less than good (%)
		Age-standardised Disability-Adjusted Life Year (DALY) rate (per 100,000 inhabitants)
		Low birth weight (%)
	Mortality	Preventable deaths (standardised death rate per 100,000 inhabitants)
		Life expectancy at birth (years)
		Infant mortality (per 1000 live births)

**Table 3:** Pairwise comparisons of the differences between metropolitan areas mean scores from the Health Determinants Index.

Group	MA	Stockholm	Athens	Barcelona	Lisbon	Berlin-Brand.	Brussels	London	Prague	Turin
1	Stockholm		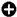	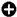	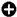	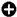	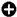	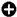	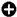	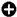
2	Athens	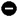		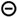	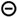	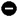	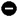	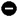	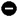	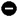
	Barcelona	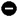	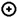		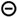	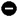	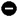	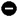	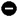	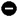
	Lisbon	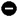	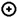	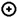		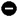	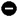	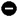	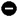	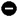
NA	Berlin-Brand.	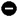	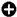	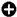	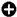		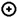	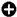	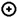	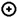
	Brussels	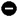	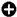	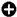	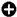	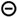		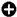	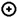	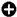
	London	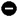	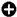	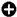	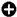	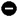	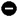		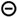	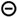
	Prague	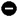	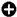	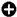	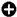	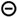	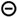	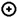		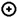
	Turin	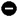	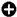	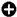	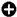	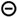	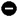	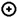	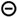	
Mean scores		87.8	58.6	61.1	62.7	77.2	76.8	70.4	73.9	73.1

Note: The symbols 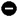 and 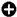 identify the metropolitan areas where scores were found to be statistically different. By way of example: Brussels presents mean scores that are statistically different from Athens, Barcelona, and Lisbon, London and Turin (with higher scores: 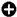) and from Stockholm (with lower scores: 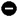). The symbols 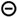 and 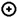 only display lower or higher differences (respectively), although not statistically significant. NA = No group was found.

**Table 4:** Pairwise comparisons of the differences between metropolitan areas mean scores from the Health Outcomes Index.

Group	MA	Stockholm	Barcelona	Turin	Lisbon	Prague	Athens	Berlin-Brand.	Brussels	London
1	Stockholm		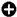	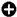	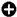	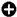	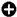	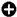	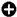	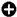
2	Barcelona	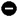		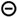	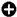	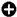	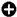	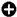	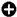	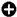
	Turin	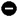	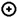		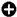	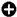	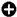	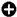	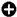	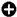
3	Lisbon	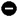	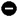	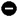		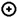	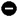	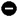	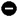	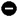
	Prague	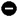	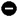	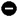	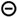		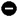	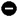	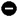	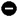
NA	Athens	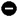	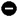	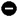	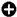	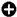		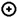	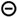	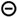
	Berlin-Brand.	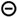	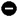	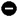	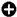	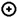	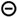		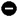	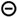
	Brussels	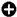	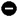	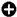	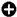	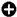	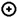	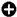		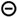
	London	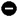	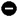	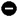	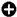	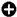	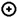	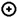	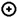	
Mean scores		94.2	82.8	83.6	66.7	66.5	74.4	72.8	76.3	76.6

Note: The symbols 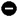 and 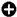 identify the metropolitan areas where scores were found to be statistically different. By way of example: Brussels presents mean scores that are statistically different from Stockholm, Turin, Lisbon, Prague and Berlin-Brandenburg (with higher scores: 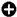) and from Barcelona (with lower scores: 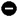). The symbols 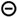 and 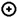 only display lower or higher differences (respectively), although not statistically significant. NA = No group was found.

## Supplementary Materials

The following are available online at https://www.mdpi.com/1660-4601/16/5/836/s1, S1: Additional information on the Local Administrative Unit and delimitation of each metropolitan area. Supplementary Materials S1 includes Table S1: Name of the administrative unit, at the Local Administrative Unit (LAU) level, Table S2: Local Administrative Unit (LAU) level and number of units, by metropolitan area, Table S3: Comparison of the delimitation of the metropolitan areas with the EUROSTAT definition and data source of the delimitation selected for this study. S2: Data availability of the indicators included in the construction of the Population Health Index by metropolitan area. Data source, year and geographical level. Supplementary Materials S2 includes Table S4: Population Health Indicators. Data source, year and geographical level, in each metropolitan area.
