# Supplementary material for: Population Health Inequalities Across and Within European Metropolitan Areas through the Lens of the EURO-HEALTHY Population Health Index
Source: Int J Environ Res Public Health. 2019 Mar 7;16(5):836. doi: 10.3390/ijerph16050836 (PMC6427561; doi:10.3390/ijerph16050836)
Supplement: Supplementary file 1 [file ijerph-16-00836-s001.pdf]

International Journal of
*Environmental Research and Public Health*

MDPI

**Supplementary Material S1:**

Additional information on the Local Administrative Unit and delimitation of each metropolitan area.

To apply the PHI to the metropolitan areas it was deemed important to identify the adequated administrative level to provide evidence on urban inequalities. For this study the definition of a municipality from the Encyclopædia Britannica was applied (http://www.britannica.com/topic/municipality): «A municipality is a political subdivision of a state within which a municipal corporation has been established to provide general local government for a specific population concentration in a defined area (…). The municipality is one of several basic types of local government, the others being counties, townships, school districts, and special districts».). However, European countries have a diversified system of local government in which several different categories exist. For instance, the term municipality may be applied in France to the *commune*, in Italy to *comune* and in Portugal to *município*.

EUROSTAT has set up a hierarchical system of Nomenclature of Territorial Units for Statistics (NUTS) and Local Administrative Units (LAUs). NUTS are organized into four levels. NUTS 0 correspond to the 28 EU countries. NUTS 1 are the major socio-economic regions within a given country. NUTS 2 are basic regions for the application of regional policies and NUTS 3 are small regions for specific diagnoses. Below, the Local Administrative Units are also organized into two hierarchical levels: The upper LAU level (LAU level 1) and the lower LAU level (LAU level 2). For most European countries, LAU-2 corresponds to municipalities, except for Bulgaria and Hungary (Settlements), Ireland (Districts), Lithuania (Elderships), Malta (Councils), Denmark (Municipalities), Greece (Municipalities), Portugal (Parishes), and the United Kingdom (Wards) [47] (Table S1). More information at: http://ec.europa.eu/eurostat/web/nuts/local-administrative-units.

**Table S1.** Name of the administrative unit, at the Local Administrative Unit (LAU) level.

| Country | LAU 1 | LAU 2 |
| --- | --- | --- |
| Belgium | Arrondissements; Verviers split into two (same as NUTS 3) | Municipalities *(Gemeenten/Communes)* |
| Czechia | Districts *(Okresy)* | Municipalities *(Obce)* |
| Germany | Collective municipalities *(Verwaltungsgemeinschaften)* | Municipalities *(Gemeinden)* |
| Spain | Provinces + Islands + Ceuta and Melilla (same as NUTS 3) | Municipalities *(Municipios)* |
| Greece | Municipalities/Communities *(Dimoi/Koinotites)* | Municipal districts/Community districts (*Demotiko diamerisma/Koinotiko diamerisma)* |
| Italy | Provinces (same as NUTS 3) | Municipalities *(Comuni)* |
| Portugal | Municipalities *(Concelhos—Municípios)* | Parishes *(Freguesias)* |
| Sweden | Counties (same as NUTS 3) | Municipalities *(Kommuner)* |
| United Kingdom | In England and Wales: Districts or individual unitary authorities<br>In Scotland: Individual unitary authorities or LECs<br>In Northern Ireland: Districts | Wards (or parts thereof) |

Source: EUROSTAT (2007). Regions in the European Union Nomenclature of territorial units for statistics. Luxembourg: Office for Official Publications of the European Communities. ISBN 978-92-79-04756-5.

Metropolitan areas specified different administrative levels for this project, according to the Local Administrative Unit (LAU) Level defined by EUROSTAT (Table S2). One metropolitan area presents a mixture of administrative levels: Berlin-Brandenburg. This metropolitan area consists of

two regions: Berlin and Brandenburg. The first enjoys a high level of power and, therefore, it has the capacity to produce local statistics to support the analysis of intra-urban disparities. Accordingly, the specified administrative level is the borough. For Brandenburg, the administrative level is the municipality.

**Table S2.** Local Administrative Unit (LAU) level and number of units, by metropolitan area.

| Metropolitan Area | LAU Level | Number of Units |
|---|---|---|
| Athens | 1 | 40 |
| Barcelona | 2 | 23 |
| Berlin-Brandenburg | 1 and 2 | 30 |
| Brussels | 2 | 121 |
| London | 1 | 33 |
| Lisbon | 1 | 18 |
| Prague | 1/2 (equal) | 57 |
| Stockholm | 2 | 26 |
| Turin | 2 | 49 |

The EUROSTAT delimitation of the metropolitan regions was taken into account to identify the geographical extent of the selected metropolitan areas. EUROSTAT defines metropolitan regions as «NUTS 3 regions, or a combination of NUTS 3 regions, which represent all agglomerations of at least 250,000 inhabitants. These agglomerations were identified using the Urban Audit's Functional Urban Area (FUA). Each agglomeration is represented by at least one NUTS 3 region. If in an adjacent NUTS 3 region more than 50% of the population also lives within this agglomeration, it is included in the metro. As the metro-regions are based on agglomerations, which include the commuter belt around a city, this approach corrects the distortions created by commuting and the GDP per inhabitant becomes meaningful, whereas comparison of GDP per inhabitant of NUTS 3 regions is far more difficult to interpret, since the difference may be partly artificial» (Source: http://ec.europa.eu/eurostat/web/metropolitan-regions/overview).

The EUROSTAT delimitation was not considered adequate by all the focal points responsible for the metropolitan areas Three metropolitan areas applied the EUROSTAT delimitation; three have taken into account national documents and the others defined their own delimitation based on functional aspects (Table S3). Moreover, two metropolitan areas used numerical cut-offs regarding population to define the municipalities being analysed: Barcelona and Prague. Table S4 presents the justification for the selection of the delimitation, as well as a map with the EUROSTAT delimitation and the one taken into consideration for this study.

**Table S3.** Comparison of the delimitation of the metropolitan areas with the EUROSTAT definition and data source of the new delimitation.

| Metropolitan area | Comparison with the EUROSTAT Delimitation of the Metropolitan Region | | | Source for the Delimitation of the Metropolitan Area | | |
|---|---|---|---|---|---|---|
| | Smaller | Equal | Larger | National Document | EUROSTAT | Functional Definition |
| Athens | ■ | | | | | ■ |
| Barcelona | ■ | | | | | ■ |
| Berlin-Brandenburg | | | ■ | | | ■ |
| Brussels | | ■ | | | ■ | ■ |
| Lisbon | | ■ | | ■ | ■ | |
| London | ■ | | | ■ | | |
| Prague | ■ | | | | | ■ |
| Stockholm | | ■ | | ■ | ■ | |
| Turin | ■ | | | | | ■ |

**Table S4.** Delimitation of the metropolitan areas: Decision, justification and difference between the EUROSTAT delimitation and the one taken into consideration for this project.

| **metropolitan area** | **Decision & Justification for the delimitation** | **Delimitation of the metropolitan area**<br>**Legend:**<br>Administrative limit according to EUROSTAT (NUTS 3, 2013 version)<br>Geographical Data delivered by the focal point (municipalities)<br>NUTS 3 (2013 NUTS version) |
|---|---|---|
| **Athens** | **EUROSTAT definition, less the remote non-urban areas**<br>• EUROSTAT comprises remote non-urban areas which are included for traditional reasons<br>• The island area has a very small population (about 70,000) spread around the various islands, very different social and economic characteristics compared to Athens. | 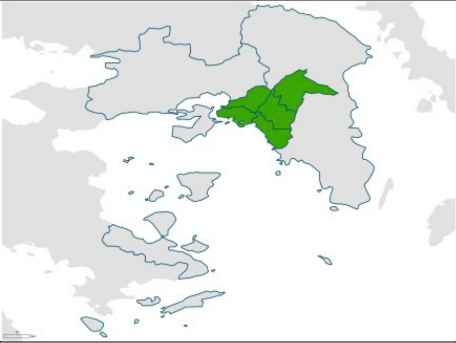 |
| **Barcelona** | **Municipalities of the Àrea Metropolitana de Barcelona with more than 10,000 inhabitants**<br>• The EUROSTAT delimitation corresponds to Barcelona Province<br>• There is an official delimitation from a public institution - Àrea Metropolitana de Barcelona - Law 31/2010 passed by the Parliament of Catalonia<br>• 13 municipalities (the farthest from Barcelona proper) have less than 10,000 inhabitants and may have issues with data availability | 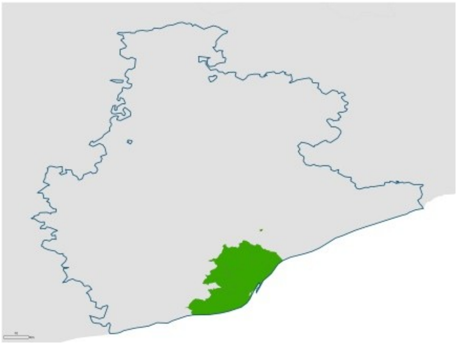 |
| **Berlin-Brandenburg** | **EUROSTAT definition plus two nearby NUTS 3**<br>• A standard definition does not exist<br>• The definition by EUROSTAT just includes Berlin and the adjoined municipalities<br>• The NUTS 3 areas DE401 "Brandenburg an der Havel" and DE403 "Frankfurt (Oder)" are two cities which have a strong relationship to the city of Berlin. e.g., "Frankfurt (Oder)" has a large university and the students usually live in Berlin and study in "Frankfurt (Oder)" | 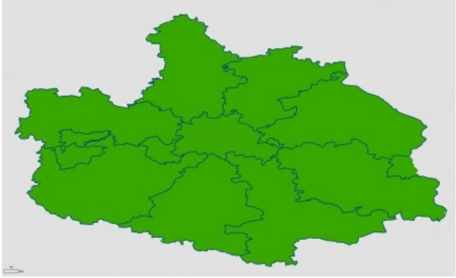 |
| **Brussels** | **Keeps EUROSTAT delimitation from the 2013 version** | 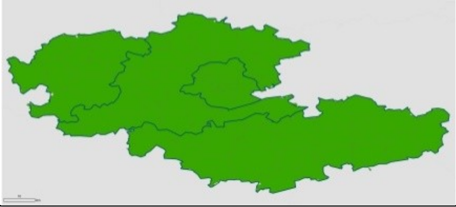 |
| **London** | **Only the NUTS 3 from Inner and Outer London**<br>• The EUROSTAT definition seems to include areas other than London NUTS for defining the metropolitan area<br>• The Office for National Statistics describes the London area very clearly | 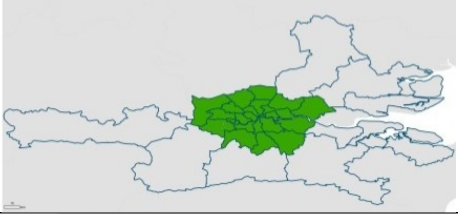 |
| **Lisbon** | **Keeps EUROSTAT delimitation**<br>• It is a public collective person of associative nature, and of territorial scope that aims to realize common public interests of the municipalities that comprise it (Law 10/2003, dated 13 May)<br>• Constituted by a public structure | 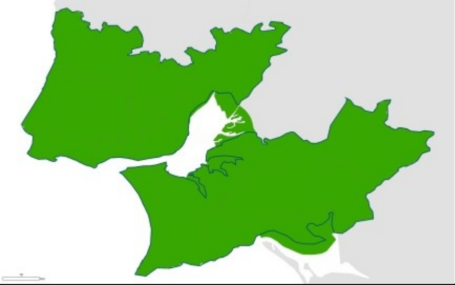 |

| metropolitan area | Decision & Justification for the delimitation | Delimitation of the metropolitan area 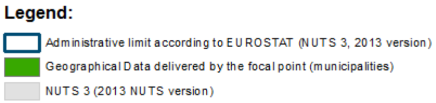  |
|---|---|---|
| **Prague** | **Municipalities with more than 5,000 inhabitants included in the NUTS 3 region CZ010 Prague**<br>• Prague administrative region is over-bounded (administrative city is larger than the geographical city) | 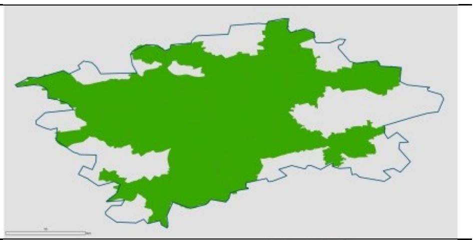 |
| **Stockholm** | **Keeps EUROSTAT delimitation**<br>• It is an official delimitation (by law) | 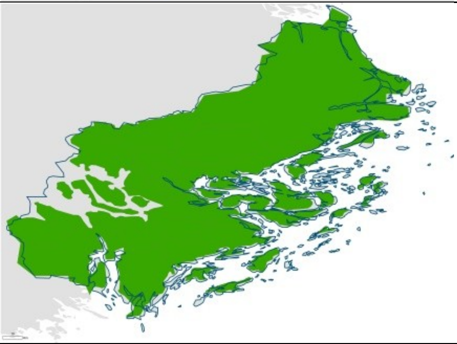 |
| **Turin** | **Combination of two different official delimitations**<br>• "Città Metropolitana di Torino"—Area Metropolitan of Turin, without the municipalities located in mountainous areas<br>• Strategic Plan of Turin—all adjacent municipalities of Turin even if located in mountainous areas | 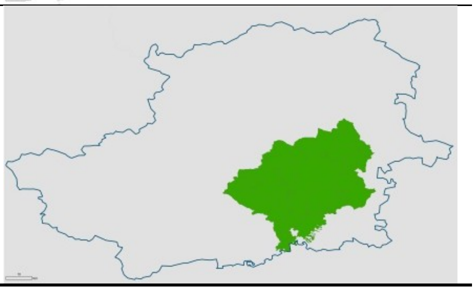 |

## Supplementary Material S2:

Data availability, data source and year of the indicators included on the construction of the Population Health Index by metropolitan area.

**Table B1.** Population Health Indicators. Data source, year and geographical level, in each Metropolitan Area.

| Indicators / Metropolitan Area | | Athens (EL) | Barcelona (ES) | Berlin-Bran-denburg (DE) | Brussels (BE) | Lisbon (PT) | London (UK) | Prague (CZ) | Stockholm (SE) | Turin (IT) |
|---|---|---|---|---|---|---|---|---|---|---|
| **COMPONENT: Health Determinants** | | | | | | | | | | |
| **AREA OF CONCERN: Economic conditions, social protection and security** | | | | | | | | | | |
| Employment | Unemployment rate (%) | ELSTAT (2011) | INE (2011) | StatIS-BBB (2014) | STATBEL (2013) | INE (2011) | ONS (2011) | CZSO (2014) | PHAS (2014) | ISTAT (2011) |
| | Long-term unemployment rate (%) | ELSTAT (2011) | IDESCAT (2014) | StatIS-BBB (2013) | EUROSTAT (2014) | INE (2011) | ONS (2011) | CZSO (2010) | PHAS (2009) | EUROSTAT (2015) |
| Income and Living conditions | Disposable income of private households PER CAPITA (Euro per inhabitant) | ELSTAT (2013) | IDESCAT (2014) | StatIS-BBB/ StatIS-BW (2013) | EUROSTAT (2012) | INE (2011) | GLA (2013) | CZSO/EU-SILC (2014) | PHAS (2012) | EUROSTAT (2013) |
| | People at risk of poverty or social exclusion (%) | EUROSTAT (2014) | EUROSTAT (2014) | EUROSTAT (2014) | EUROSTAT (2011) | EUROSTAT (2014) | EUROSTAT (2014) | EUROSTAT (2014) | EUROSTAT (2014) | EUROSTAT (2014) |
| | Disposable income ratio—S80/S20 | EUROSTAT (2013) | EUROSTAT (2014) | EUROSTAT (2014) | EUROSTAT (2014) | EUROSTAT (2014) | EUROSTAT (2014) | EUROSTAT (2014) | EUROSTAT (2014) | EUROSTAT (2014) |
| Social protection | Expenditure on care for the elderly (% of GDP) | EUROSTAT (2008) | EUROSTAT (2008) | EUROSTAT (2008) | EUROSTAT (2008) | EUROSTAT (2008) | EUROSTAT (2008) | EUROSTAT (2008) | EUROSTAT (2008) | EUROSTAT (2008) |
| Security | Crimes recorded by the police (per 100,000 inhabitants) | EH/EURO STAT (2010) | EH/EURO STAT (2010) | EH/EURO STAT (2010) | EH/EURO STAT (2010) | EH/EURO STAT (2010) | EH/EURO STAT (2010) | EH/EURO STAT (2010) | EH/EURO STAT (2010) | EH/EURO STAT (2010) |
| **AREA OF CONCERN: Education** | | | | | | | | | | |
| Education | Population aged 25–64 with upper secondary or tertiary education attainment (%) | ELSTAT (2011) | INE (2011) | StatIS-BBB (2013) | STATBEL (2011) | INE (2011) | ONS (2011) | CZSO (2011) | PHAS (2014) | ISTAT (2011) |
| | Early leavers from education and training (%) | ELSTAT (2011) | IDESCAT 2014 | StatIS-BBB (2013) | STATBEL (2011) | INE (2011) | ONS (2011) | EUROSTAT (2015) | PHAS (2012) | ISTAT (2011) |
| **AREA OF CONCERN: Demographic change** | | | | | | | | | | |
| Ageing | At risk of poverty rate of older people (%) | EUROSTAT (2014) | EUROSTAT (2014) | EUROSTAT (2014) | EUROSTAT (2014) | EUROSTAT (2014) | EUROSTAT (2014) | EUROSTAT (2014) | EUROSTAT (2014) | EUROSTAT (2014) |
| | Ageing index (ratio) | ELSTAT (2011) | IDESCAT (2014) | StatIS-BBB (2014) | STATBEL (2015) | INE (2015) | ONS (2014) | CZSO (2014) | SCB (2013) | ISTAT (2011) |
| **AREA OF CONCERN: Lifestyle and health behaviours** | | | | | | | | | | |
| Lifestyle and health | Adults who are obese (%) | HHF (2014) | INE (2011) | StatIS-BBB (2013) | Sciensano (2013) | INE (2014) | NHS (2006) | IHIS (2014) | PHAS (2013) | ISTAT (2013) |

| Indicators / Metropolitan Area | | Athens (EL) | Barcelona (ES) | Berlin-Brandenburg (DE) | Brussels (BE) | Lisbon (PT) | London (UK) | Prague (CZ) | Stockholm (SE) | Turin (IT) |
|---|---|---|---|---|---|---|---|---|---|---|
| behaviours | Daily smokers—aged 15 and over (%) | HHF (2014) | INE (2011) | StatIS-BBB (2013) | Sciensano (2013) | INE (2014) | NHS (2014) | NIPH (2014) | PHAS (2013) | ISTAT (2013) |
| | Pure alcohol consumption—aged 15 and over (Litres per capita) | HFA-DB (2011) | HFA-DB (2010) | HFA-DB (2013) | HFA-DB (2012) | HFA-DB (2011) | HFA-DB (2012) | HFA-DB (2013) | HFA-DB (2011) | HFA-DB (2010) |
| | Live births by mothers under age of 20 (%) | ELSTAT (2013) | IDESCAT (2014) | DESTATIS (2014) | EUROSTAT (2013) | INE (2010–2014) | ONS (2014) | IHIS (2013) | NBHW (2001) | CSI PIEMONTE (2006–2010) |
| **AREA OF CONCERN: Physical environment** | | | | | | | | | | |
| Pollution | Annual mean of the daily $PM_{2.5}$ concentrations (ug/$m^3$) | EH/HOOG/ESCAPE (2010) | EH/HOOG/ESCAPE (2010) | EH/HOOG/ESCAPE (2010) | EH/HOOG/ESCAPE (2010) | EH/HOOG/ESCAPE (2010) | EH/HOOG/ESCAPE (2010) | EH/HOOG/ESCAPE (2010) | EH/HOOG/ESCAPE (2010) | EH/HOOG/ESCAPE (2010) |
| | Annual mean of the daily $PM_{10}$ concentrations (ug/$m^3$) | EH/HOOG/ESCAPE (2010) | EH/HOOG/ESCAPE (2010) | EH/HOOG/ESCAPE (2010) | EH/HOOG/ESCAPE (2010) | EH/HOOG/ESCAPE (2010) | EH/HOOG/ESCAPE (2010) | EH/HOOG/ESCAPE (2010) | EH/HOOG/ESCAPE (2010) | EH/HOOG/ESCAPE (2010) |
| | Greenhouse Gas (total tonnes of $CO_2$ eq. emissions per capita) | EUROSTAT (2013) | EUROSTAT (2013) | EUROSTAT (2013) | EUROSTAT (2013) | EUROSTAT (2013) | EUROSTAT (2013) | EUROSTAT (2013) | EUROSTAT (2013) | EUROSTAT (2013) |
| **AREA OF CONCERN: Built environment** | | | | | | | | | | |
| Housing conditions | Average number of rooms per person | ELSTAT (2011) | INE (2011) | DESTATIS (2014) | STATBEL (2011) | INE (2011) | ONS (2011) | CZSO (2011) | EUROSTAT (2013) | ISTAT (2011) |
| | Households without indoor flushing toilet (%) | ELSTAT (2011) | INE (2011) | DESTATIS (2011) | STATBEL (2001) | INE (2011) | ONS (2011) | CZSO (2011) | EUROSTAT (2011) | EUROSTAT (2011) |
| | Households without central heating (%) | ELSTAT (2011) | INE (2011) | DESTATIS (2011) | STATBEL (2011) | INE (2011) | ONS (2011) | CZSO (2011) | EUROSTAT (2011) | EUROSTAT (2011) |
| Water and sanitation | Population connected to public water supply (%) | ELSTAT (2011) | EUROSTAT (2010) | 2010 | EUROSTAT (2009) | INE (2009) | EUROSTAT (2009) | CZSO (2015) | SCB (2014) | 2011 |
| | Population connected to wastewater treatment plants (%) | ELSTAT (2011) | EUROSTAT (2012) | 2013 | VMM (2015) | INE (2009) | EEA (2010) | CZSO (2015) | SCB (2014) | EUROSTAT (2005) |
| Recycling | Recycling rate of municipal waste (%) | EUROSTAT (2013) | ARC (2014) | EUROSTAT (2014) | EUROSTAT (2013) | INE (2014) | GLA/EUROSTAT (2014–2015) | EUROSTAT (2014) | Avfall Sverige (2014) | CSI PIEMONTE (2014) |
| **AREA OF CONCERN: Road safety** | | | | | | | | | | |
| Road safety | Victims in road accidents—injured and killed (per 100,000 inhabitants) | ELSTAT (2011) | SCT (2014) | StatIS-BBB (2014) | STATBEL (2014) | INE (2014) | TfL (2014) | CZSO (2014) | STA (2013) | RPG (2014) |
| | Fatality rate due to road traffic accidents (per 1000 victims) | ELSTAT (2014) | SCT (2014) | StatIS-BBB (2014) | STATBEL (2014) | INE (2014) | TfL (2014) | CZSO (2014) | NBHW (2013) | RPG (2014) |
| **AREA OF CONCERN: Healthcare resources and expenditure** | | | | | | | | | | |
| Healthcare resources | Medical doctors (per 100,000 inhabitants) | ELSTAT (2013) | IDESCAT (2014) | ÄK/KVBB (2014) | EUROSTAT (2012) | GEOHEALTHS (2014) | NHS (2014) | IHIS (2013) | EUROSTAT (2013) | CSI PIEMONTE (2015) |

| Indicators / Metropolitan Area | | Athens (EL) | Barcelona (ES) | Berlin-Brandenburg (DE) | Brussels (BE) | Lisbon (PT) | London (UK) | Prague (CZ) | Stockholm (SE) | Turin (IT) |
|---|---|---|---|---|---|---|---|---|---|---|
| | Health personnel—nurses and midwives, dentists, pharmacists and physiotherapists (per 100,000 inhabitants) | ELSTAT (2011) | IDESCAT (2014) | StatIS-BBB (2011) | EUROSTAT (2012) | EUROSTAT (2015) | NHS (2014) | IHIS (2013) | EUROSTAT (2013) | CSI PIEMONTE (2015) |
| Healthcare expenditure | Total health expenditure (PPP$ per capita) | HFA-DB (2013) | HFA-DB (2013) | HFA-DB (2013) | HFA-DB (2013) | HFA-DB (2013) | HFA-DB (2013) | HFA-DB (2013) | HFA-DB (2013) | HFA-DB (2013) |
| | Private households' out-of-pocket expenses on health (% total health expenditure) | HFA-DB (2013) | HFA-DB (2013) | HFA-DB (2013) | HFA-DB (2013) | HFA-DB (2013) | HFA-DB (2013) | HFA-DB (2013) | HFA-DB (2013) | HFA-DB (2013) |
| | Total health expenditure (PPP$ per capita) | HFA-DB (2013) | HFA-DB (2013) | HFA-DB (2013) | HFA-DB (2013) | HFA-DB (2013) | HFA-DB (2013) | HFA-DB (2013) | HFA-DB (2013) | HFA-DB (2013) |
| **AREA OF CONCERN: Healthcare performance** | | | | | | | | | | |
| Healthcare performance | Hospital discharges due to diabetes, hypertension and asthma (per 100,000 inhabitants) | EH/EURO STAT (2011) | EH/EURO STAT (2013) | EH/EURO STAT (2013) | EH/EURO STAT (2012) | EH/EURO STAT (2014) | EH/EURO STAT (2011) | EH/EURO STAT (2013) | EH/EURO STAT (2010) | EH/EURO STAT (2013) |
| | Amenable deaths due to health care (standardized death rate per 100,000 inhabitants) | EH/ELSTAT (2009–2013) | INE (2009–2013) | StatIS-BBB (2009–2013) | STATBEL (2007–2011) | INE (2009–2013) | ONS (2009–2013) | CZSO (2009-2013) | SCB (2009–2011) | 2009–2012 |
| **COMPONENT: Health Outcomes** | | | | | | | | | | |
| **AREA OF CONCERN: Health Outcomes** | | | | | | | | | | |
| Morbidity | Self-perceived health less than good (%) | EUROSTAT (2014) | EUROSTAT (2014) | EUROSTAT (2014) | EUROSTAT (2014) | EUROSTAT (2014) | EUROSTAT (2014) | EUROSTAT (2014) | EUROSTAT (2014) | EUROSTAT (2014) |
| | Age-standardized Disability-Adjusted Life Year (DALY) rate (per 100,000 inhabitants) | HFA-DB (2012) | HFA-DB (2012) | HFA-DB (2012) | HFA-DB (2012) | HFA-DB (2012) | HFA-DB (2012) | HFA-DB (2012) | HFA-DB (2012) | HFA-DB (2012) |
| | Low birth weight (%) | ELSTAT (2009–2013) | IDESCAT (2010–2013) | StatIS-BBB/ DESTATIS 2009–2013 | STATBEL (2004–2008) | INE (2007–2011) | PHOF (2009–2013) | IHIS (2009–2012) | NBHW (2012) | CSI PIEMONTE (2011–2015) |
| Mortality | Preventable deaths (standardized death rate per 100,000 inhabitants) | EH/ELSTAT (2009–2013) | INE (2009–2013) | StatIS-BBB (2009–2013) | STATBEL (2007–2011) | INE (2009–2013) | ONS (2009–2013) | CZSO (2009–2013) | SCB (2009–2011) | 2009–2012 |
| | Life expectancy at birth (years) | EurOhex (2013) | ASPB (2013) | StatIS-BBB (2012) | EUROSTAT (2013) | INE (2012-2014) | ONS (2012-2014) | CZSO (2010-2014) | SCB (2012) | ISTAT/ CSI PIEMONTE (2012) |
| | Infant mortality (per 1000 live births) | ELSTAT (2009–2013) | INE (2010–2013) | StatIS-BBB (2010–2014) | EUROSTAT (2009–2013) | INE (2010–2014) | ONS (2009–2013) | IHIS (2008–2012) | PHAS (2012–2014) | CSI PIEMONTE (2006–2010) |

Note: More information about how the indicators where built is available on the Atlas of Population Health European Regions [31].

Legend: Green = available at municipal level; yellow = available at a geographical level that allows the identification of some inequality (e.g., district); red = available at regional or national level). Abbreviations: ÄK = Berlin Medical Association; ARC = Catalan Waste Agency; ASPB = Barcelona Public Health Agency; CSI PIEMONTE = Information System Consortium—Piemonte Region; CZSO = Czech Statistical Office; DESTATIS = Federal Statistical Office of Germany; EEA = European Environment Agency; EH = Euro-Healthy Project; ELSTAT = Hellenic Statistical Authority; ESCAPE = European Study of Cohorts for Air Pollution Effects; EurOhex = European Health Expectancy Monitoring Unit; EUROSTAT = European Statistical Office; EU-SILC = European Union Statistics on Income and Living Conditions; GEOHEALTHS = Geography of health status—An application of a Population Health Index in the last 20 Years; GLA = Greater London Authority; HHF = Hellenic Health Foundation; HFA-DB = European Health for All database; HOOG = de Hoogh K et al. (2016) Development of west-european $PM_{2.5}$ and $NO_2$ land use regression models incorporating satellite-derived and chemical transport modelling data, Environmental Research, 151: 1–10; IDESCAT = Official Statistics of Catalonia; IHIS = Institute of Health Information and Statistics of the Czech Republic; INE (ES) = National Statistics Institute; INE (PT) = Statistics Portugal; ISTAT = Nationla Statistics Institute; KVBB = Brandenburg Medical Association; NBHW = National Board of health and welfare; NHS = National Health Service; NIPH = National Institute of Public Health (Czech Republic); PHAS = Public Health Agency Sweden; PHOF = Public Health Outcomes framework; RPG = Piemonte Regional Government; SCB = Statistics Sweden; Sciensano = Belgian institute for health; SCT = Catalan Traffic Service; STA = Swedish Transport Agency; STATBEL = Statistics Belgium; StatIS-BBB = Statistical Office Berlin-Brandenburg; StatIS-BW = Statistical Office Baden-Wuerttemberg; TfL = Transport for London; VMM = Flanders' regional environmental agency.
